# Low Zinc Status and Absorption Exist in Infants with Jejunostomies or Ileostomies Which Persists after Intestinal Repair

**DOI:** 10.3390/nu4091273

**Published:** 2012-09-12

**Authors:** Kimberly S. Balay, Keli M. Hawthorne, Penni D. Hicks, Zhensheng Chen, Ian J. Griffin, Steven A. Abrams

**Affiliations:** 1 Black Hills Pediatrics & Neonatology, LLP, 2905 5th Street, Rapid City, SD 57701, USA; Email: ksbalay@gmail.com; 2 USDA/ARS Children’s Nutrition Research Center, Baylor College of Medicine, 1100 Bates Street, Houston, TX 77030, USA; Email: keli.hawthorne@bcm.edu (K.M.H.); penni.hicks@bcm.edu (P.D.H.); zhensheng.chen@bcm.edu (Z.C.); 3 UC Davis Medical Center, University of California, 2516 Stockton Blvd, Sacramento, CA 95817, USA; Email: ijgriffin@ucdavis.edu

**Keywords:** zinc, copper, ostomy, jejunostomy, ileostomy, nutrient absorption

## Abstract

There is very little data regarding trace mineral nutrition in infants with small intestinal ostomies. Here we evaluated 14 infants with jejunal or ileal ostomies to measure their zinc absorption and retention and biochemical zinc and copper status. Zinc absorption was measured using a dual-tracer stable isotope technique at two different time points when possible. The first study was conducted when the subject was receiving maximal tolerated feeds enterally while the ostomy remained in place. A second study was performed as soon as feasible after full feeds were achieved after intestinal repair. We found biochemical evidence of deficiencies of both zinc and copper in infants with small intestinal ostomies at both time points. Fractional zinc absorption with an ostomy in place was 10.9% ± 5.3%. After reanastamosis, fractional zinc absorption was 9.4% ± 5.7%. Net zinc balance was negative prior to reanastamosis. In conclusion, our data demonstrate that infants with a jejunostomy or ileostomy are at high risk for zinc and copper deficiency before and after intestinal reanastamosis. Additional supplementation, especially of zinc, should be considered during this time period.

## 1. Introduction

Gastroschisis, other abdominal wall defects, and conditions such as necrotizing enterocolitis are common in a neonatal intensive care (NICU) setting. Many infants with these conditions require long-term total parenteral nutrition (TPN) and some may require the placement of a small bowel ostomy, usually a jejunostomy or ileostomy. The provision of enteral nutrition, including trace mineral nutrition, in these infants can be difficult due to impaired nutrient absorption. However, details of intestinal absorptive function in this time period are not available and very few studies have specifically evaluated it.

The evaluation of trace mineral status, especially zinc and copper, in high-risk infants, including those with ostomies, is extremely difficult. Studies of serum zinc and copper are difficult to perform and interpret in this population, in part due to the need for a relatively large blood volume for biochemical analysis. Balance studies of zinc absorption and retention are very difficult to perform in this group using mass balance techniques due to the challenges of multi-day collections of all urine and stool. However, the use of stable isotope technology to measure mineral bioavailability using the dual-tracer method and the use of high precision ICP technology to evaluate total minerals on extremely low blood volumes makes such studies more feasible.

Although zinc deficiency is the more likely clinical concern in these conditions, the interaction of zinc and copper is such that excess of zinc might affect copper status. Intestinal metallothionein binding and saturation is closely involved in regulation of both copper and zinc absorption and biochemical status. Therefore, optimally investigations of zinc include some measure of copper status as well. 

Inadequate zinc status may be related to poor intake, inadequate absorption fraction or increased excretory losses in infants with stomas. Our objective in this study was to evaluate these possible causes of zinc deficiency and determine if deficiencies in trace mineral status and poor zinc bioavailability were present in an unselected population of neonatal intensive care unit infants with small intestinal ostomies. We hypothesized that we would identify evidence of poor zinc and copper status and reduced zinc absorption when receiving nearly full feeds, both before and after, intestinal repair and reanastamosis. 

## 2. Methods

### 2.1. Study Population

Infants admitted to the NICU of Texas Children’s Hospital, Houston, TX, who had a jejunostomy or ileostomy were recruited for the study. The study was approved by the Institutional Review Board of Baylor College of Medicine and Affiliated Hospitals. Neonates were enrolled if they had a jejunostomy or ileostomy and if their birth weight was >500 g. Infants were excluded from the study if they had any other major congenital anomalies (including congenital heart disease and cystic fibrosis), or if they were believed to be unlikely to survive to hospital discharge based on their cardiopulmonary disease. 

### 2.2. Study Overview

Seventeen subjects were recruited between September 2008 and May 2010. The first part of the study was conducted while the ostomy was in place. It consisted of a measurement of biochemical zinc and copper status. Zinc absorption and excretion evaluations were performed as soon as TPN was stopped and the infant was receiving 100–140 mL/kg/day of enteral intake. This timing was used as venous access is often discontinued in our patients within 24 h of stopping TPN. However several infants did reach that goal and were studied while still receiving some TPN. A second study was conducted once the intestine had been reconnected and the infant was receiving full enteral feels. Zinc absorption, but not zinc excretion, was measured in the second study.

Ten infants participated in first study (prior to reanastamosis), twelve in the second study (after reanastamosis), and eight infants participated in both studies. Eight of the 10 infants who participated in the first study also took part in the second study. Four infants who had not done the first study as they did not achieve >50% feeds prior to anastomosis, took part in the second study. Therefore, overall, 14 of the 17 recruited subjects completed at least one study (both studies *n* = 8; first study only *n* = 2; second study only *n* = 4).

### 2.3. Data Collection

Birth weight, gestational age at birth, corrected gestational age during the study, gender, race, reason for jejunostomy or ileostomy, amount of bowel resected, location of bowel resected, presence or absence of ileocecal valve, and date of reanastamosis surgery were recorded. 

Nutrition information was collected including the volume and composition of parenteral nutrition, intravenous lipids, and enteral feeds. We calculated the calories and zinc intake via the parenteral and enteral routes separately, and the total nutrient intake from both routes combined. Urine, ostomy, and replogle tube output volume were also measured. 

Serum zinc and serum copper were determined in our lab using a high-resolution inductively coupled plasma mass spectrometry ICP-MS (Bremen, Germany). Serum ceruloplasmin was measured by standard clinical techniques.

### 2.4. Zinc Metabolic Methods

The stable isotope methods for assessing zinc absorption and endogenous excretion have been published in detail previously, and extensive details are available elsewhere [1]. Briefly, zinc fractional absorption was measured using a dual tracer stable isotope method in which ^67^Zn was given orally with a single feed followed immediately by infusion of ^70^Zn intravenously. A spot urine sample was collected 96 h after the infusion and the relative dose-corrected enrichments used to calculate fractional absorption at the time oral isotope was administered. 

Tracer:tracee ratios (TTR), measured by ICP-MS, were used to calculated fractional zinc absorption using the equation:


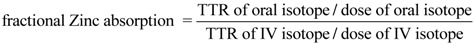


Endogenous fecal Zn excretion was measured from the urinary and fecal excretion of the intravenously administered tracer and the urinary excretion of tracee Zn in a 4-day urine and fecal collection using the equation:


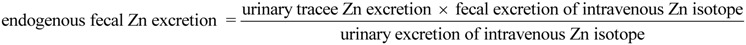


Zn balance (retention) was estimated from the equation:


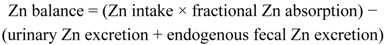


Zinc intake is expressed as mg/kg/day. Zinc absorption is expressed in fraction (% of intake) and in absolute (mg/kg/day) terms. Fecal zinc excretion, urinary zinc excretion, and zinc balance are expressed as mg/kg/day.

### 2.5. Statistical Analysis

Standard descriptive statistics (e.g., mean ± SD) are used. Relationships between zinc absorption, zinc excretion (urine or fecal) and zinc balance, and potential explanatory variables were analyzed using the GLM model function of JMP 7 for Macintosh (SAS Inc., Cary, NC, USA). Simple and multiple regression analysis were used as appropriate. Significance was assumed at a *p* < 0.05.

## 3. Results

### 3.1. Study #1—Prior to Reanastamosis

#### 3.1.1. Subject Demographics

Ten subjects took part in the isotope study with an ostomy (Table 1).

**Table 1:** Demographic data of the subjects.

Characteristics	Study #1	Study #2
Number	10	12
Gestational Age (weeks)	25.8 ± 3.2	27.2 ± 4.0
Birth weight (g)	873 ± 473	1070 ± 574
Post-natal age (days)	75 ± 25	114 ± 23
Post-menstrual age (weeks)	36.6 ± 5.5	43.3 ± 6.7
Weight (g)	2045 ± 756	3022 ± 881
Length (cm)	42.3 ± 4.6	49.2 ± 4.8
Fronto-occipital circumference (cm)	30.0 ± 3.0	34.3 ± 2.4
Gender	2M:8F	4M:8F
Disease	7, isolated perforation	7, isolated perforation
	1, volvulus	1, volvulus
	1, NEC	3, NEC
	1, meconium plug	1, meconium plug
Bowel resection	None = 5	None = 4
	Small bowel = 4	Small bowel = 6
	Small and large bowel = 1	Small and large bowel = 2
Small bowel resection?	5 Y:5 N	8 Y:4 N
Ileocecal valve intact?	10 Y:0 N	11 Y:1 N

Seven infants were studied when they were on full enteral feeds, and three were studied on less than full enteral feeds immediately prior to reanastamosis. Of those not receiving all their nutrition enterally, all three were receiving 1 g/kg/day intravenous lipid, and they were receiving approximately 40, 60 or 80 mL/kg/day of parenteral nutrition. The site of the ostomy was not always clearly defined by the surgical team, but the ileocecal value was reported intact in all but one infant. An average of 9 cm of bowel was reported removed with only one infant having >30 cm of bowel removed.

Biochemical results from the time of the two study periods are reported in Table 2. 

**Table 2:** Biochemical data at the time of study.

Parameter	Study #1	Study #2
Sodium	134 ± 3 (*n* = 10)	137 ± 3 (*n* = 12)
Potassium	5.0 ± 0.9 (*n* = 10)	4.7 ± 0.6 (*n* = 12)
Chloride	103 ± 4 (*n* = 10)	103 ± 3 (*n* = 12)
Bicarbonate	24 ± 4 (*n* = 10)	26 ± 5 (*n* = 12)
BUN	13.6 ± 7.6 (*n* = 7)	14.7 ± 5.1 (*n* = 7)
Creatinine	0.48 ± 0.42 (*n* = 4)	0.28 ± 0.04 (*n* = 6)
Calcium	9.7 ± 0.6 (*n* = 9)	10.0 ± 0.6 (*n* = 7)
Phosphorus	5.9 ± 0.5 (*n* = 9)	6.3 ± 0.4 (*n* = 7)
Magnesium	2.2 ± 0.2 (*n* = 3)	2.1 ± 0.1 (*n* = 5)
Alkaline Phosphatase	361 ± 153 (*n* = 9)	420 ± 142 (*n* = 9)
Unconjugated bilirubin	0.9 ± 0.7 (*n* = 10)	0.9 ± 0.4 (*n* = 7)
Conjugated bilirubin	1.2 ± 2.2 (*n* = 10)	1.3 ± 1.3 (*n* = 9)
Serum zinc	896 ± 358 (*n* = 10)	1072 ± 357 (*n* = 11)
Serum copper	599 ± 394 (*n* = 10)	756 ± 360 (*n* = 11)
Serum ceruloplasmin	14.5 ± 4.6 (*n* = 10)	16.8 ± 6.8 (*n* = 12)

Serum zinc was less than 750 μg/L in 5 subjects (Table 2) none of whom were receiving TPN. Serum copper was less than 500 μg/L in 5 subjects, one of whom was receiving TPN. Serum ceruloplasmin was less than 20 mg/dL in 9 subjects and it was above 20 mg/dL in only a subject, who was receiving TPN. There was no significant correlation between serum zinc and serum copper (*r*^2^ = 0.26, *p* = 0.074), or between serum zinc and serum ceruloplasmin (*r*^2^ = 0.11, *p* = 0.35). 

#### 3.1.2. Nutrient Intakes

Total zinc intake averaged 1.22 ± 0.57 mg/kg/day of which the majority (1.13 ± 0.56 mg/kg/day) was enteral and relatively little (0.10 ± 0.17 mg/kg/day) was from parenteral sources in the three infants still receiving some TPN. Total copper intake was 162 ± 84 μg/kg/day, of which 156 ± 89 μg/kg/day was enteral and 7 ± 13 μg/kg/day was parenteral. 

#### 3.1.3. Fractional Zinc Absorption

Fractional zinc absorption was 10.9% ± 5.3% of zinc intake. It was unaffected by gender (*p* = 0.11), small bowel resections (*p* = 0.15), enteral zinc intake (*p* = 0.94) but was inversely related to serum zinc (*p* = 0.034). Three infants had an elevated conjugated bilirubin (conjugated bilirubin >1.5 mg/dL). There was no relationship (*p* > 0.5) between conjugated bilirubin level and absorption fraction or other measures of zinc status. 

#### 3.1.4. Absolute Zinc Absorption

Absolute zinc absorption was 0.18 ± 0.06 mg/kg/day. It was unaffected by gender (*p* = 0.18), small bowel resection (*p* = 0.21), but was significantly positively correlated with enteral zinc intake (*p* = 0.0154), and tended to be negatively correlated with serum zinc (*p* = 0.055).

#### 3.1.5. Zinc Excretion

Endogenous fecal zinc excretion (0.19 ± 0.21 mg/kg/day) was significantly greater in males (*p* = 0.036), was lower in those with small bowel resections (*p* = 0.011) and in those on higher enteral zinc intake (*p* = 0.032) It also tended to be lower at higher serum zinc levels (*p* = 0.052).

Urinary zinc excretion (0.05 ± 0.05 mg/kg/day) was lower in males (*p* = 0.099), in those on higher enteral zinc intakes (*p* = 0.011), and in those with lower serum zinc levels (*p* = 0.002). It was unaffected by small bowel resections (*p* = 0.28).

#### 3.1.6. Zinc Balance

Net zinc balance was not significantly different from zero (−0.027 ± 0.24 mg/kg/day, *p* = 0.75). Three subjects were in negative zinc balance, and 7 were in positive zinc balance. Net zinc balance trended to be better in the three infants still receiving parenteral nutrition (0.137 ± 0.094 mg/kg/day) than in the seven who were on full enteral feeds (−0.097 ± 0.256 mg/kg/day, *p* = 0.068).

Relatively higher net zinc balance was seen in females (*p* = 0.012), those with small bowel resections (*p* = 0.0006), with higher enteral zinc intakes (*p* = 0.0012), and higher serum zinc levels (*p* = 0.0026).

### 3.2. Study #2—after Reanastamosis

#### Subject Demographics

Twelve subjects were studied on full enteral feeds after reanastamosis, just prior to hospital discharge (Table 1 and Table 2). There was an average of 17 ± 8 days between reanastamosis and this second study. Three of the 12 infants had a serum zinc <750 μg/L, five had a serum copper below 500 μg/L and ten had a ceruloplasmin <20 mg/dL.

Zinc intake averaged 1.06 ± 0.78 mg/kg/day and was not closely related to gender (*p* = 0.71), small bowel resections (*p* = 0.72), enteral zinc intake (*p* = 0.65) or serum zinc (*p* = 0.23). Zinc absorption was 9.3% ± 5.7%. 

Among the 8 subjects who had zinc absorption measured at both time points, zinc absorption fraction did not change significantly between the two time periods (10.6% ± 5.9% *vs.* 9.9% ± 6.2%, *p* = 0.82). Fractional zinc absorption was unaffected by gender (*p* = 0.57) or small bowel resections (*p* = 0.18) but was higher at lower zinc intakes (*p* = 0.006), and tended to be higher at lower serum zinc concentrations (*p* = 0.059). 

## 4. Discussion

In this study we identified a substantial number of children who had, or had recently had an ostomy with evidence of zinc or copper deficiency. These deficiencies are likely caused by poor mineral absorption as we demonstrated for zinc. After intestinal reanastamosis, there was still evidence of zinc malabsorption, although zinc status appeared improved, likely in part because our study was performed soon after stopping TPN. Taken together, these data suggest that enteral feeds provided during refeeding may not provide adequate zinc and possibly copper to meet infants’ needs. 

Zinc deficiency is a major concern in both small preterm infants and especially infants who have mineral losses secondary to bowel damage. Clinically, zinc deficiency can be manifest in multiple ways including increases in infection and poor growth and appetite. Zinc supplmentation is provided to at-risk infants, but in our nurseries this is only done using intravenous nutrition; oral zinc supplements in any form are not used. Excess zinc supplementation could occur and be manifest by copper deficiency which would lead to hematological abnormalities or bone abnormalities, but this is likely to be uncommon.

Our study is the first to use stable isotopes or to evaluate zinc status in a group of infants with small intestinal ostomies. The use of isotope based studies and ICP analysis of total minerals allows for a very small amount of blood to be used and only a single infusion of a zinc isotope intravenously. This approach is safe and led to the acceptance of the research protocol by parents who might otherwise decline being involved in research without direct benefit for their infant. 

Defining biochemical zinc deficiency is difficult in the clinical neonatal intensive care setting. We have arbitrarily used a value of 750 μg/L as evidence of deficient status, recognizing that this is not well-established [2]. In our infants, when on TPN, the mean serum zinc (*n* = 7) was 1074 μg/L and only one infant who was unable to ever be fed had evidence of severe deficiency. Zinc intakes averaged 1.2 mg/day and as all zinc IV was absorbed, and zinc requirements for growth are probably about 400 μg/day in preterm infants [3,4], it is not surprising that zinc status was adequate with this intake. 

Ultimately, only 10 infants achieved a majority of enteral feeds with the ostomy in place, of which only 7 were fully enterally fed at the time of the study. This provided a mixed group, receiving a range of feeding types, but nonetheless representative of infants with small intestinal ostomies. What was remarkable is that all 10 infants showed poor zinc absorption, averaging only about 10% absorption. 

The data from Jalla *et al.* [3] in preterm infants without ostomies, fed either fortified human milk or preterm formula are best used for comparison. These data suggest that the primary problem is one of intestinal malabsorption, not increased endogenous secretion of zinc. None of these infants had their ostomy outputs refed. Comparison of results with those from mass balances are not easily done due to technical differences. However, low intakes led to negative balance in those studies as well [5] (Table 3). 

**Table 3:** Zinc balance data in prior studies compared to current study.

	Jalla (*n* = 14) [3]	Loui (*n* = 10) [5]	Current (*n* = 7)
Dietary intake (μg/day)	2680	780	2510
Absorption (%)	26	-	11.5
Endogenous Secretion (μg/day)	280	-	315
Net Retention (μg/day)	400	−200	−100

After the infants had their gastrointestinal tracts reconnected, it was not possible to do a full balance study involving fecal collections. However, we were able to conduct a dual tracer zinc isotope study measuring zinc absorption fraction as well as obtain serum values. These studies were done as soon as the infant had reached full feeds so as to take advantage of any indwelling central line before it was removed. Nonetheless, values at this time for zinc absorption remained low. Net retention could not be calculated without the endogenous secretion values, but based on intakes of about 3 mg/day, and endogenous fecal values from Jalla *et al.* [3] and from the earlier part of this study, it is likely that subjects were in negative or only very marginally positive balance at this time. Serum zinc values were better, with only 3 infants below 750 μg/L. These results suggest a gradual improvement after reanastamosis, with some concern about low zinc status and low copper status in the initial refeeding period.

## 5. Conclusions

In summary, we have shown that infants with a small bowel ostomy, regardless of diagnosis or enteral intake are at high risk for zinc deficiency, primarily during the time in which they still have an ostomy but are being fed enterally and extending at least into the early refeeeding period post-ostomy. From our limited data, the primary appears to be poor absorption of dietary zinc. Very high secretory losses were not seen in our subjects and may not reflect the largest etiology of inadequate zinc status. This explains in part why zinc status remains good while on TPN with extra zinc but not after TPN is stopped. Although very high levels of oral or intravenous zinc could be provided, this would pose its own risks due to osmolar concerns and lack of well tested products, especially in the United States.

Controlled trials of zinc supplementation are warranted, although care will be needed to ensure that the forms provided are safe and bioavailable. It may also be useful to evaluate increasing zinc contents of formulas or human milk fortifiers used for this population. 
